# Cases of Surgery

**Published:** 1845-05

**Authors:** Daniel Brainard

**Affiliations:** Prof. of Surgery in the Rush Medical College


*Cases of Surgery.* By Daniel Brainard, M. D., Prof. of Surgery in the Rush Medical College.

CASE I.

*Extirpation of Submaxillary Gland.*—B. T., of Ottawa, Ills., æt. about 38 years, consulted me in relation to a tumor of the lower jaw.

*Present state.*—On examination, I found a tumor extending from near the angle forward three inches on the jaw, upward to a level with the inferior molar teeth, and downward deeply into the neck, beneath the base of the jaw. With the finger in the mouth it could be felt involving the submaxillary gland. It was very hard and firm, except at the most projecting point, where it was elastic, and gave the sensation of fluctuation.

*History.*—The patient gave the following account of its developement. Seven months since (in August, 1844,) he had a decayed tooth, upon the right side of the lower jaw, which was extracted; soon after which, a small tumor was perceived upon the outside, below the point of its situation. This, when perceived, was of the size of a bean, immoveable (he thinks), very firm and little painful. It continued to increase until it attained the size we have described; its progress not having been checked by the use of iodine, blisters, &c., which had been resorted to for that purpose.

*Treatment.*—In order to remove all doubt, a small puncture of exploration was made, at the fluctuating point, from which no pus, but only a small quantity of redish serum, was discharged. The removal of the tumor was then determined upon and performed, March 17th, 1845, with the assistance of Drs. Herrick and Blaney, of this city, Dr. Abbott, of La Salle co., and in presence of several students of medicine, in the following manner:

An incision was made of a semi-lunar form, commencing at the side of the chin, extending along the base of the jaw, and terminating in front of the ear. Two others were then made upon the side of the neck, in such a manner, as with the first, to circumscribe a triangular space, embracing the most projecting part of the tumor. These would have enabled us, if it had been found necessary, to remove a portion of the jaw. The dissection was then continued from the mucous membrane of the mouth, a task of some delicacy, from the side of the jaw to which the growth was adherent, but from which it could be separated. The dissection was then commenced below, and the profound attachments of the tumor were exposed. The *cornu.* of the *os hyodes*, the nerve of the ninth pair, the *milo-hyoid* muscle, the lingual branch of the fifth nerve, were successively brought into view, and, finally, its connections, which were intimate with the membrane of the mouth, at the side of the tongue, were separated and its removal thus completed. This dissection was extremely tedious, as well from the numerous arterial branches, among which were the facial and lingual branches of the carotid, requiring ligatures, as from the proximity of this latter vessel and the internal jugular vein, which it was desirable not to wound. At this stage of the operation, it was found that several lymphatic glands were diseased, whose intimate connections with the great vessels of the neck, rendered their removal a matter of considerable difficulty. This was at length effected, however, by the aid of the handle of the scalpel. Considerable blood was lost, but the patient bore the operation well. Simple dressings were applied, and the cicatrization of the wound proceeding favorably, the patient was enabled, on the 26th March, to leave for home, under the charge of Dr. Abbott, his medical attendant.

*Examination of the Tumor.*—This was found, when laid open, to consist of a tissue evidently scirrhus, softened at the point where it had been punctured, involving the submaxillary and several lymphatic glands.

CASE II.

*Extirpation of an Encephaloid Tumor from the Neck.*—C. F. consulted me, April 7th, 1845, with a tumor situated upon the right side of the neck, a little below the angle of the jaw, upon the anterior margin of the *sterna-mastoid* muscle. It was of the size of a large hickory nut, very firm, adherent to the skin above and to the muscle beneath. The patient had first perceived it about five weeks previously, and its growth had been attended with slight pains. It was removed the same day; the operation presented nothing unusual, if we except a tendency to hæmorrhage, which rendered necessary the application of three ligatures, and compression, to suppress the oozing of blood over the whole surface. Nothing unfavorable occurred, and the patient was able to leave for Michigan on the 11th of April. On laying open the tumor, it was found to consist at the outside of a tissue like scirrhus, but at the centre contained a quantity of substance, of the size of a large bean, having all the characters of encephaloid tissue.

*Remarks.*—Both the above cases are illustrative of a fact not sufficiently known, namely: that cancerous tumors, in their development, are often unattended by pain of any kind; and the latter case is an example, somewhat rare, of the deposition of the cerebriform matter in the tumor of moderate size.

